# 581. COVID-19 Vaccine Perceptions in Adults from Greater Nashville Tennessee

**DOI:** 10.1093/ofid/ofab466.779

**Published:** 2021-12-04

**Authors:** Kailee N Fernandez, Danielle A Rankin, Harrison L Howe, Sean M Bloos, Seifein Salib, Rana Talj, Herdi Kurnia Rahman, Danya Waqfi, Jessica Villarreal, Ahmad Yanis, Leigh Howard, James Chappell, Natasha B Halasa, Natasha B Halasa

**Affiliations:** 1 Vanderbilt University Medical Center; Division of Pediatric Infectious Diseases, Nashville, Tennessee; 2 Vanderbilt University Medical Center, Goodlettsville, Tennessee

## Abstract

**Background:**

In December 2020, SARS-CoV-2 vaccines were made available to healthcare workers and soon thereafter offered to the general public according to age and risk of severe illness. Despite widespread access, vaccination rates vary by region, with Tennessee ranking lower than the national average. Therefore, we aimed to survey adults in greater Nashville, TN regarding SARS-CoV-2 vaccine perceptions.

**Methods:**

We conducted a cross-sectional study of an ongoing longitudinal cohort of individuals with confirmed and/or suspected SARS-CoV-2 infection and their household contacts with enrollment onset in March 2020. For this analysis, individuals were included if they were ≥ 18 years and available for a one-year follow-up visit. At the one-year visit individuals completed a survey about vaccine preferences, beliefs and risks. Demographic and social characteristics were collected at enrollment. Individuals were considered vaccinated if they had received at least one dose of a SARS-CoV-2 vaccine under FDA emergency use authorization. Vaccine perceptions were compared by SARS-CoV-2-infection and vaccination status using Pearson’s chi-squared, alpha=5%.

**Results:**

Between April-May 2021, 115 individuals completed the one-year follow-up. Table 1 includes sociodemographic characteristics of adults, of which the majority were vaccinated and were unemployed or in non-essential occupations. Most individuals agreed the SARS-CoV-2 vaccine can prevent infection and hospitalization (Figure 1A & B). Unvaccinated participants more often agreed that those who contracted SARS-CoV-2 should not receive the vaccine (30%), whereas vaccinated persons less often agreed (11%, p< 0.001) (Figure 1A). Additionally, 44% of unvaccinated individuals were neutral or disagreed that benefits of SARS-CoV-2 vaccination outweighed the illness risk, compared to 10% in the vaccinated group, p=0.001 (Figure 1A). Minimal differences of vaccine perceptions were observed between SARS-CoV-2 positive and negative adults (Figure 1B).

Table 1. Sociodemographic Characteristics of Adults

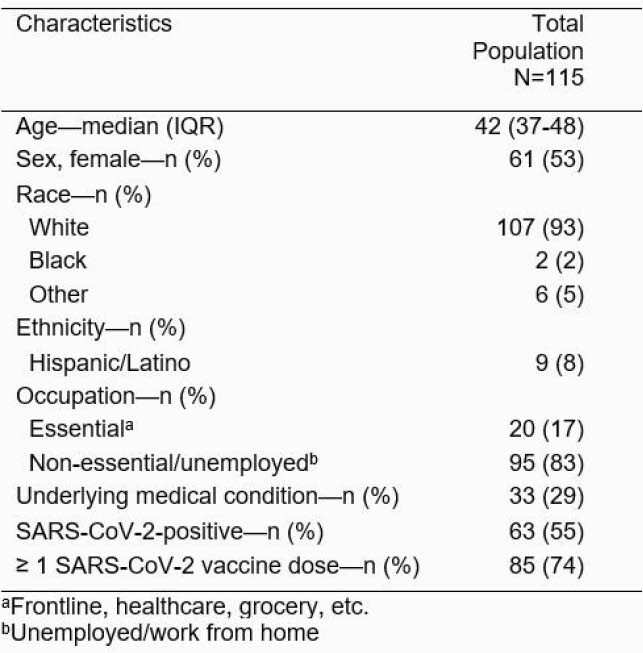

Figure 1. Vaccine perceptions of vaccinated and unvaccinated (A) SARS-CoV-2 positive and SARS-CoV-2 negative (B) adults in greater Nashville, TN. Vaccine perceptions were collected through a standardized survey at the one-year visit.

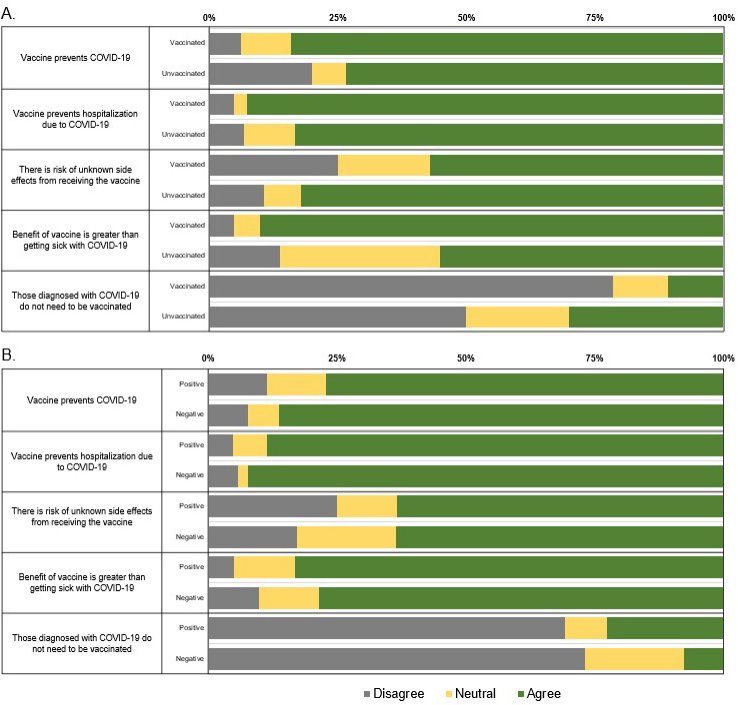

**Conclusion:**

Although some unvaccinated individuals seemingly perceived the SARS-CoV-2 vaccine offered some protection, research should continue to evaluate the implications of vaccine hesitancy on the COVID-19 pandemic response as we prepare for the upcoming respiratory season.

**Disclosures:**

**Natasha B. Halasa, MD, MPH**, **Genentech** (Other Financial or Material Support, I receive an honorarium for lectures - it's a education grant, supported by genetech)**Quidel** (Grant/Research Support, Other Financial or Material Support, Donation of supplies/kits)**Sanofi** (Grant/Research Support, Other Financial or Material Support, HAI/NAI testing) **Natasha B. Halasa, MD, MPH**, Genentech (Individual(s) Involved: Self): I receive an honorarium for lectures - it's a education grant, supported by genetech, Other Financial or Material Support, Other Financial or Material Support; Sanofi (Individual(s) Involved: Self): Grant/Research Support, Research Grant or Support

